# Inclusive Strategies for Children With Developmental Disabilities in Mainstream Classrooms in African Countries: A Systematic Review of Stakeholder Experiences, Attitudes, and Perspectives

**DOI:** 10.3102/00346543241288247

**Published:** 2024-10-31

**Authors:** Elisa Genovesi, Akhina Gaches, Judith McKenzie, Charlotte Hanlon, Rosa A. Hoekstra

**Affiliations:** King’s College London; University of Cape Town; King’s College London; Addis Ababa University; King’s College London

**Keywords:** inclusive education, teaching strategies, developmental disabilities, Africa

## Abstract

African children with developmental disabilities (DD), including autism and intellectual disability, are often excluded from mainstream schools. We systematically reviewed 28 qualitative studies conducted in Africa, aiming to synthesize stakeholders’ perspectives on inclusive teaching strategies for supporting children with DD in mainstream classes. Six interrelated themes were developed using thematic analysis. Teachers strived to meet learners’ needs through teaching and task adaptations (Theme 1) and provide targeted support, reinforcement, and feedback (Theme 2). Teachers’ inclusive pedagogies, while sometimes creating divisions, often reflected a whole-class approach (Theme 3), especially in promoting inclusive environments (Theme 4) and engaging teaching (Theme 5). Authors of reviewed studies often discussed how teachers’ strategies fit in with the evidence base (Theme 6). While teachers’ inclusion efforts appeared progressive, they were mostly based on Western pedagogies and challenged by limited resources and training. Training efforts based on indigenous pedagogies could improve use and outcomes of inclusive strategies.

In line with the United Nations’ Convention on the Rights of Persons With Disabilities (UN General Assembly, 2007), several African countries are committed to promoting inclusive education, initially promoted internationally by the Salamanca Statement (United Nations [UN], 1994), which states that education should be provided to all “within the regular education system” through “a child-centred pedagogy” (UN, 1994). Additionally, in this article, we specifically define inclusive education as the practice of addressing the needs of all learners in mainstream classes (UNESCO Global Education Monitoring Report Team, 2020): This means that children with disabilities included in the mainstream education system are not segregated in separate classes within mainstream schools, and that their inclusion in mainstream classes is not a mere physical placement in the class; rather, it involves an attention to their additional needs on the part of the educator. The relevant literature presents controversies on whether these needs should be addressed only through whole-class approaches, such as generally inclusive environments and pedagogies, or also through special education strategies, such as individualized accommodations of the needs of children with disabilities (Francisco et al., 2020). However, our definition of inclusive education does not limit this aspect, as *how* the needs of children with disability are addressed in mainstream class is the topic of our enquiry.

Despite African governments’ commitment to inclusive education, a large proportion of children with disabilities in Africa remain excluded from education (World Bank, 2018), due to factors such as stigma in the community and within school settings, poor building accessibility, and limited availability of trained personnel and of materials for inclusive teaching (Genovesi et al., 2022; UNESCO Global Education Monitoring Report Team, 2020). Among children with disabilities, systematic exclusion is reportedly higher for those with developmental disabilities (DD) (Ajuwon & Chitiyo, 2016; Andrews at al., 2020; Genovesi et al., 2022; UNESCO Global Education Monitoring Report Team, 2020), defined as lifelong conditions characterized by difficulties in social, communication, and/or cognitive skills that manifest in early childhood, such as intellectual disability, autism, and attention-deficit hyperactivity disorder (ADHD) (World Health Organization, 2016).

## The Context of the Study: Non-Arab African Countries

Africa is a highly diverse continent, in terms of history, economy, and culture. One key cultural distinction that can be made is between Arab countries, mostly inhabited by Arabic-speaking Muslim populations and with several cultural similarities with Middle Eastern countries, and non-Arab countries. In this report, we focus on the second group and define it as the group of 48 countries the World Bank (2023) refers to as “sub-Saharan Africa.” However, we refrain to use this term, considered geographically inaccurate and ideologically problematic (e.g., De Haldevang, 2016), and use the term “African” for brevity, while acknowledging that we are not referring to all countries on the African continent.

Distinct from Arab countries, these countries have a predominance of Christian faiths or African Native religions, and the region presents a plethora of indigenous languages. Of the 48 countries in this group, only the Seychelles are classed as high-income, with the other countries included ranging from low- to upper-middle-income (World Bank, 2023). The World Bank (2023) also highlights economic development challenges, despite the large availability of natural resources, often due to Western control of those natural resources for decades in throughout the colonial history of most countries in this region (Murombedzi, 2016). The region’s economic growth has also been slowed down in recent years due to conflicts in several countries, climate change, and increasing debts with Western countries, although growth rates diverge across countries (World Bank, 2023).

While African countries share some similarities in their social structures, cultures, and/or economic and political challenges that contrast them to Arab countries, their high internal and cross-country diversity must also be acknowledged. The 48 countries present an extraordinary richness of ethnicities, languages, religions, and customs and differences in their economics and politics that may reflect on their education systems. Nonetheless, scientific evidence also shows similarities in terms of resource constraints and in attitudes, practices, and challenges relevant to including children with DD in government schools (Genovesi et al., 2022; UNESCO Global Education Monitoring Report Team, 2015).

In a recent systematic review of qualitative studies in African countries (Genovesi et al., 2022), we synthesized stakeholders’ experiences of inclusive education for children with DD and their perspectives on barriers and opportunities for including children with DD in mainstream schools. The 32 studies included were conducted in South Africa, Zimbabwe, Botswana, Ghana, Uganda, Nigeria, and Eswatini and explored the experiences of teachers, parents, and children, with some studies including multiple stakeholders. The synthesis highlighted relevant factors at the community, school, class, and individual levels, which were common to studies conducted in different settings. Key common barriers reported were unclear or poorly implemented education policies and low political commitment to inclusion in all countries of the included studies, and insufficient training, resources, and support for teachers. A striking everyday challenge for teachers was caused by large class sizes of 40 to 80 students under the responsibility of a single teacher, who therefore found it hard to address the needs of children with DD. At the same time, the review identified opportunities for inclusion across studies, such as teachers’ commitment to inclusion, collaboration between teachers, and the work of nongovernmental organizations (NGOs), typically very active in the region. Included studies reported that teachers often strived to promote an inclusive environment in the classroom, where children with DD were welcomed and supported by their peers and the physical environment was conducive to every pupil’s learning. Studies also reported that some teachers encountered challenges in addressing the needs of children with DD and managing their behavior, with some using corporal punishment, as reported in two Ghanaian studies (Okyere et al., 2019a, 2019b). A large portion of data discussed the use of pedagogical strategies for inclusion, such as adaptations, cooperative work, and reinforcement. Data from study participants and discussions from the authors included nuanced reflections on the extent to which these strategies were in line with education theories—often derived from Western high-income countries—or indigenous perspectives of inclusion, or how they helped address issues or were hindered by contextual challenges such as limited human and material resources. The appropriateness, use, and helpfulness of such strategies was a prominent and multifaceted theme that we were unable to analyze and discuss in sufficient depth in our previous synthesis (Genovesi et al., 2022). Nonetheless, these reflections were crucial, as interventions focused on pedagogy in general can be highly effective in improving students’ performance in African countries (Conn, 2017). Specifically, identifying inclusive education pedagogies that teachers in some African countries can use and deem acceptable within their contexts can inform dissemination of effective strategies and teachers’ professional development programs in other African countries. Indeed, while the diversity of the countries may require appropriate adaptation and contextualization of any imported practices, pedagogies from one African country are likely to be relevant to others that may share similar perspectives and challenges (Genovesi et al., 2022). Thus, such evidence can promote the dissemination of practices originating from the African context itself, rather than of Western pedagogies that may be less feasible, acceptable, or useful in African countries. We take up this aspect in the current article, presenting an in-depth synthesis on perspectives and experiences on the use of pedagogical strategies in inclusive classrooms in African countries, aimed at capturing the nuance of discussions on challenges, facilitators, and benefits of such strategies in the articles included in our previous review (Genovesi et al., 2022).

In international education theory, the relevance of teaching strategies is derived from the idea, championed by authors in the field of Disabilities Studies in Education, that inclusive education is not limited to the physical placement of children with disabilities in mainstream classes; rather, it is about providing an inclusive learning experience in which all children participate actively (Baglieri, Bejoian, et al., 2011). Notably, while the field of Disabilities Studies in Education originated in high-income countries, it has been previously reflectively applied to the African context, including by African authors ( J. A.McKenzie & Dalton, 2020). Genovesi et al. (2022) also reported aligned African stakeholders’ definitions of inclusive education, such as “commitment to enhance the achievement of all children” (Majoko, 2018, p. 642). Inclusion in this sense requires a teaching approach that promotes the participation of all children in the learning experience. Disabilities Studies in Education envision this approach as one in which barriers to learning are removed for all learners, with and without disabilities. This is consistent with the Social Model of Disability claim that disabilities are the product of barriers posed by social structures, rather than inevitable consequences of physiological impairments (Baglieri, Bejoian, et al., 2011; Baglieri & Shapiro, 2012; Baglieri, Valle, et al., 2011). Within the context of education, this translates into a shift in attention from remediating individual deficits, towards the creation of accessible learning environments that enable learner achievement (Slee, 2019). Therefore, an analysis of strategies used in a context, in this case African countries, can shed a light on broader local understandings of disability, inclusion and pedagogy, including the relevance of Disability Studies in Education to these perspectives: Such understanding can then have implications for the development of more context-responsive developments in African pedagogy, inclusive education, and disability inclusion more broadly.

Consequently, we set to analyze approaches and strategy selection and use in African countries and reflections by study participants and authors. We aimed to understand local models of inclusion rooted in African cultures and contexts, as well as informing dissemination in African countries of practices considered acceptable and effective in the included studies from this context. In doing so, we acknowledge the value of local stakeholders’ perspectives in inclusive education (UNESCO Global Education Monitoring Report Team, 2020), as in any innovation and development in education (Penuel et al., 2020) and public health (Damschroder et al., 2009). Local stakeholders have, in fact, a key role in uncovering the local understanding of theoretical and aspects of the innovations and elicit context-appropriate problem solving for barriers that may hinder their implementation (Damschroder et al., 2009; Penuel et al., 2020).

We have explored the following research question: What stakeholders’ experiences and perspectives on inclusive classroom strategies for children with DD are evident in Africa, with regard to their use, aims, contextual appropriateness, benefits, disadvantages and alignment with accepted conceptualizations of inclusion?

## Method

The current article reports a secondary analysis of a systematic review aimed at exploring stakeholder perspectives on inclusive education for children with DD in Africa (Genovesi et al., 2022). Specifically, the reported analysis focused on extracts discussing the use of inclusive strategies in the classroom.

### Systematic Search and Study Selection

The methods of the original review are reported elsewhere (Genovesi et al., 2022), following the *enhancing transparency in reporting the synthesis of qualitative research* (ENTREQ) statement (Tong et al., 2012) and including a *PRISMA* flow diagram (Moher et al., 2009). Here we provide a summary of the key steps. Detailed information on the full search terms and inclusion and exclusion criteria can be found online in Supplementary File A in the online version of the journal. The review protocol was pre-registered on *PROSPERO* (National Institute for Health Research, n.d.) prior to the database search (protocol CRD42020185486). Peer-reviewed journal articles were searched in PsycInfo (Ovid), MEDLINE (Ovid), Embase (Ovid), Global Health (Ovid), and ERIC (Ebsco), combining four concepts:

1. experiences, attitudes, perspectives, and generally qualitative data;
2. education;
3. DD (intellectual disabilities, autism, ADHD, language and social communication disorders, undiagnosed developmental delays) and related concepts (including “disability” and “special needs”); and
4. countries in the World Bank group “sub-Saharan Africa” (World Bank, 2023).

The search yielded 6,051 results after deduplication. No date limits were imposed in the database search or selection criteria to avoid limiting the comprehensiveness of the review. Relevant studies were selected through consensus between two independent researchers using a two-stage screening process: (a) title-and-abstract screening to identify potentially relevant studies and (b) selection of studies which met all criteria through a full-text assessment of 172 potentially relevant studies. Disagreements were resolved by a third researcher. Further relevant studies were identified through forward and backward citation checks of all studies selected.

We included qualitative studies on stakeholder experiences, perspectives, and attitudes towards inclusive education for pupils with DD in primary and secondary schools in Africa, intended as children with DD learning in mainstream classes. We excluded studies on special school education, not relevant to the aims of our review (e.g., Ellman et al., 2020) and quantitative studies on attitudes to inclusive education for children with DD (e.g., Al Shoura & Ahmad, 2020), as we aimed to explore in-depth qualitative descriptions in stakeholders’ own words. When the studies had a broader scope on disability inclusive education, we included those where at least 40% of participants were children with DD, or teachers/caregivers of children with DD, as suggested by participant or setting information, to ensure sufficient relevance to our aims. For example, a report of Special Needs Education teacher trainees’ perceptions of inclusive education in Zimbabwe (Chireshe, 2013) was excluded because it focused on inclusive education in general, with no specific focus on DD, and no indication of participants specializing in DD.

The quality of the studies selected was appraised by two reviewers using the Critical Appraisal Skills Programme (CASP; 2018) checklist for qualitative studies.

### Synthesis Approach and Positionality

The Results and Discussion sections of each study report were synthesized thematically (Thomas & Harden, 2008) in the original review. During this process, extracts relevant to the use of inclusive strategies were selected for the present synthesis. These were analyzed in NVivo-12 through reflexive thematic analysis (Braun & Clarke, 2019), with the aim to develop inductive themes focused on a central concept or shared meaning, such as functions and features of the strategies used, rather than categories or types of different strategies (or “domain summary themes”; see Braun and Clarke [2019] for a discussion). Data were further analyzed to explore connections among themes and subthemes and develop a richer account through a thematic map. In line with a critical realist approach (Bhaskar, 2017), the researchers aimed to capture subjective stakeholder experiences of an existing reality. In line with the aims of Disabilities Studies in Education as a discipline described by Baglieri, Bejoian, et al. (2011), the analysis did not focus on the most technical aspects of the strategies employed by teachers, such as details on specific tasks, needs assessment or timings. Rather, we interrogated the main approaches, the common drives to selecting particular strategies, and the overall aims of such practices, striving to understand how these fit in terms of the teachers’, participants’, and authors’ cultural models of inclusion and social justice.

EG conducted the analysis in collaboration with AG, with the aim to explore meaning in greater depth through discussion and iteratively revise codes accordingly. Rather than eliminating subjectivity, such discussions allowed for conscious exploration of positionality and subjective interpretations, in line with the chosen analytical approach (Braun and Clarke, 2019). The researchers who conducted the analyses (EG and AG) have in common with the majority of the participants in the included studies (particularly teachers and caregivers) that they are women. However, they identify as outsiders in relation to the communities studied. As researchers in the UK, broadly identifying as White European and middle-class, they were mindful throughout the analysis of cultural and socioeconomic differences with such communities and often with researchers of the studies reviewed. The first author, EG, identifies with aspirations to achieve a truly equitable and inclusive society, and her work aims to promote fully inclusive education systems, while acknowledging that this may not be an easily implementable endeavor in low-resourced settings. EG’s higher education background is in psychology, particularly neurodiversity and global mental health. She has some practical work experience in education settings and knowledge of education theory, and she has visited several special and mainstream schools in one African country, Ethiopia. AG, whose background is also in psychology with some education work experience, allowed EG to reflexively recognize assumptions originating from education theory. JMK, CH, and RAH have academic backgrounds in inclusive education, global mental health, and global autism research, respectively, and they have extensive knowledge of various African countries where they are based ad/or work and where they have visited schools. All authors had in common a shared positive attitude towards inclusive education and its implementation in low-resource settings.

## Synthesis

Twenty-eight of the 32 papers in the original review (Genovesi et al., 2022) included references to inclusive pedagogical strategies. These papers are signaled with an asterisk (*) in the reference list of this report. The relevant studies were conducted between 2001 and 2020 in South Africa (13), Zimbabwe (5), Botswana (4), Ghana (3), and Uganda (3). Some studies had multiple participants groups; others focused on one group. Overall, participants were teachers (25 studies), parents (4 studies) and pupils with DD (8 studies) and without DD (2 studies). Participants were typically recruited from inclusive schools. Except for three studies of poorer quality, the reviewed quality appraisals of the research ranged from average to excellent, meaning that they fulfilled between 6 and 10 of the CASP checklist points on the aims, design, methods, and relevance of the study (CASP, 2018). The most common weakness was insufficient reporting on some methodological aspects, such as how the number of participants was established or the authors’ positionality. Additional study features are reported in Supplementary File C in the online version of the journal.

The study reports differed in the extent to which they focused on teachers’ practice, with 5 studies including only one or two short relevant extracts (Bannink, Nalugya, et al., 2020; P. S.de Jager, 2011; Engelbrecht et al., 2001; Mapuranga et al., 2015; Seabi, 2010), 5 discussing a whole theme on one or more pedagogical strategies (Lopes et al., 2009; Majoko, 2015, 2018, 2019; Yoro et al., 2020), 4 being entirely focused on this topic (Majoko, 2017; Mangope, 2017; Mokobane, 2011; Otukile-Mongwaketse et al., 2016), and the 14 fourteen including few but substantial extracts. Therefore, not all studies are equally represented in the synthesis.

**Figure 1.**
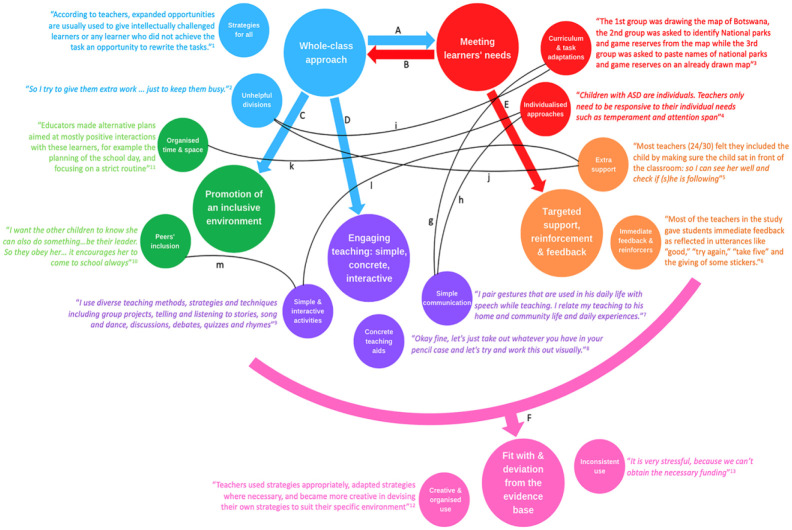
*Thematic map of stakeholders’ perspectives and experiences of inclusive strategies used in African school classrooms to include children with developmental disabilities.* Subthemes are exemplified by extracts from synthesized papers, with direct quotes from participants in Italics. Citations: (1) Lopes et al., 2009; (2) Mokobane, 2011; (3) Otukile-Mongwaketse et al., 2016; (4) Majoko, 2015; (5) Bannink et al., 2016; (6) Mangope, 2017; (7) Majoko, 2018; (8) Walton and Rusznyak, 2014; (9) Majoko, 2017; (10) Okyere et al., 2019b; (11) Van Schalkwyk and Marais, 2017; (12) Potgieter-Groot et al., 2012; (13) Engelbrecht et al., 2003. Full references are available in the reference list. See Supplementary File B in the online version of the journal for full explanations of the connections highlighted by the thematic map.

Our interpretation of the data lead to the development of six interdependent themes, each with subthemes, as shown in the thematic map in Figure 1. As shown by black lines in the figure, subthemes were closely interrelated across themes as well.

In the synthesis below, themes are exemplified by quotes from the studies (in inverted commas), which in turn often report participants’ words (in italics). While some studies and participants focused on strategies as specifically applied to children with DD, many discussed their use for learners identified as weaker, regardless of presence or absence of DD. The language used in this synthesis reflects this diversity. While in reflexive thematic analysis theme relevance is independent from the number of data items mentioning the theme, in this review synthesis we provide some quantitative frequency information to give a more detailed description of the evidence reviewed.

### Theme 1: Meeting Learners’ Needs

It was apparent in several studies that teachers applied a key inclusive education principle: the responsiveness to a diverse range of learner needs, through curriculum and task adaptations and individualized instruction and selection of strategies.

#### Subtheme 1.1: Curriculum and task adaptations

While participating teachers were not always able “to adapt teaching and learning content to the individual level of cognitive functioning” (Majoko, 2019), this was deemed a key skill for inclusive teaching by several participants and authors of the studies reviewed.

Curriculum adaptations, here intended specifically as adapting the taught content, include teaching those concepts and skills that students are able and need to acquire according to teachers. In the studies reviewed, the inclusion of learners with DD who struggled to carry out day-to-day activities and interact with others required teaching such skills as part of the curriculum. Two studies on including children with autism (Majoko, 2017, 2018) and one on those with emotional and behavioral challenges (Potgieter-Groot et al., 2012), reported teachers directly instructing learners on social skills. Other life skills added to the curriculum to the benefit of children with DD included “combing hair and tying shoelaces” (Majoko, 2017), “*brushing the teeth*” (Mangope, 2017), fine motor skills in manipulating objects ( P. S.de Jager, 2011), and “projects like bee keeping” (Mukhopadhyay et al., 2019). However, it was often unclear how the skills to be included in the curriculum were selected for individual learners.

A key component of curriculum adaptations was task differentiation: assigning diversified activities to different (groups of) students, with some teachers ensuring that these were “focusing on the same objective” (Mangope, 2017) of the curriculum. Thus, while the task varied, the intended outcome for all students did not. At times, teachers created groups of children according to their abilities, to facilitate task differentiation for every group, as observed by Otukile-Mongwaketse et al. (2016):

The 1st group was drawing the map of Botswana, the 2nd group was asked to identify National parks and game reserves from the map while the 3rd group was asked to paste names of national parks and game reserves on an already drawn map.

Additionally, task differentiations were implemented during assessments, to promote accessibility and achievement. Assessment adaptations also entailed “expanded opportunities” (Mokobane, 2011), such as teachers’ support and the possibility to retake an assessment, and “alternative response modes” (Majoko, 2017). The latter included oral, rather than written, assessment (Walton & Rusznyak, 2014; Yoro et al., 2020), or the opportunity for learners with communication challenges to respond through drawings (Majoko, 2017; Walton & Rusznyak, 2014). When describing similar adaptations, participants stressed their role in ensuring that all learners had opportunities for achievement.

#### Subtheme 1.2: Individualized approaches

Researchers and teacher participants often stressed that curriculum adaptations and other strategies should be centered around the individual learner. For example, when using reinforcement as a pedagogical strategy, reinforcers can be selected to meet learners’ own interests (Majoko, 2017). Similarly, children with DD may vary in their need for a highly structured environment, and teachers should account for this variation when deciding how much structure to provide to learners (Majoko, 2015).

*Children with ASD are individuals. Teachers only need to be responsive to their individual needs such as temperament and attention span when approaching their teaching and learning.* (Majoko, 2015)

Addressing individual needs is the core aim of individualized plans, which were said to be key to make inclusive teaching effective. While in some studies, adaptations seemed based on general assumptions about DD, as highlighted above, in others it was emphasized how adaptations should be targeted specifically to learners’ needs but also to their existing skills and knowledge. For example, teachers in two studies assessed learners’ entry competencies to plan for appropriate curriculum adaptations (Majoko, 2017, 2018).

### Theme 2: Targeted Support, Reinforcement, and Feedback

A set of individualized strategies used by teachers in inclusive education involved providing learners with support and feedback targeted to their needs during teaching and tasks.

#### Subtheme 2.1: Extra support

Teachers recognized that children with learning and behavioral difficulties need extra individualized attention. This could involve checking on them closely in class “*to ensure they are on task*” (Majoko, 2017) and they don’t “*slip away and end of up being naughty*” (Lopes et al., 2009). Another purpose was to strengthen the learning of children who needed extra support to understand lesson content or complete tasks.

Teachers implemented multiple strategies to provide extra attention. For example, several teachers in a study in South Africa highlighted how grouping children in class per ability allowed them to know “*where to focus more*” (Yoro et al., 2020). From the analysis of relevant extracts, it appeared that such groups were mere seating clusters, aimed at quickly recognizing students’ level, rather than group activities. “Strategic seating arrangements” (Majoko, 2017) were also mentioned as an effective strategy to “minimize distractions” (Seabi, 2010).

Most teachers (24/30) felt they included the child by making sure the child sat in front of the classroom: *so I can see her well and check if she is following*. (Bannink et al., 2016)

Finally, remedial classes outside of the school timetable were used to support learners’ progress individually or in smaller groups of learners for whom teachers adapted the curriculum and implemented “improved strategies such as mind maps, reading and writing exercises” (Yoro et al., 2020).

#### Subtheme 2.2: Immediate feedback and reinforcers

When observed in their practice and when interviewed, teachers in several studies showed to be “aware of the importance of giving reinforcement or feedback” (Mangope, 2017), and especially immediate feedback, to promote learners’ motivation and support their learning. Feedback was aimed at keeping students engaged and helping them correct themselves. It was provided through general exclamations, such as “good” or “try again” (Mangope, 2017) as well as more specific support in identifying mistakes (Mokobane, 2011).

Similarly, reinforcers, including verbal praise, preferred activities and play, preferred objects and toys, and other tokens and rewards (e.g. stickers, sweets), were used to motivate children. For example, one teacher, talking about a child with DD said:

*I feel so happy when he is able to understand one little thing and get the answer correct . . . so I give him toffees to encourage him to do more*. (Okyere et al., 2019a)

Reinforcers were also used beyond school tasks, to reward learners’ positive behavior and, in the case of two studies on the inclusion of children with autism, socially appropriate behavior. This strategy helped them manage learners’ aggressive or disruptive behavior and support their learning of social skills.

### Theme 3: Whole-Class Approach

Together with meeting individual learners’ needs, the evidence base for inclusive education recommends that strategies are implemented with a whole-class approach (e.g. Norwich & Lewis, 2013), to support effective inclusion by targeting the needs of all children and refraining from making specific distinctions between children with and without DD, “*to avoid labeling and stigmatization*” (Majoko, 2017). A Ugandan study highlighted that this approach may be especially valuable in collectivistic African cultures which merge individual’s identities with their communities, where “individual needs should not be ignored, but addressed within a larger framework of ‘we’ as a class” (Bannink, Nalugya, et al., 2020).

#### Subtheme 3.1: Strategies for all

Most of the inclusive strategies discussed in the studies were, at times, used to the benefit of all learners. There were two main ways in which teachers reported applying strategies to all learners. First, methods such as teaching social skills were applied to everyone in the same way. Others, such as adaptations and simplified instructions, were applied to each learner in the classroom differently, in accordance with individual needs of each learner in the class, but without making clear distinctions between children with and without disabilities. For example, teachers often discussed how simplified instruction and tasks met the needs of “weak” or “slow” learners, rather than just those with identified as having a DD.

Participants were observed providing individualized instruction and support including coaching and prompting to both learners with and without autism who had baseline skills and competencies. (Majoko, 2017)

#### Subtheme 3.2: Unhelpful divisions

Exploring the central concept of whole-class approaches in our analysis, instances in which this principle was neglected in the name of adaptations or extra support to children with DD became apparent in Zimbabwe, South Africa, Uganda, and Botswana. In practice, teachers were not always able to treat all pupils equally. The authors of the study reports reviewed highlighted how some of the strategies used for task/curriculum differentiation and to give extra attention to children with learning and behavioral difficulties could foster discrimination of the same learners, when they were applied segregating children with DD, rather than addressing each learner’s needs.

Workloads could be greater for children with DD compared to their peers for several reasons, including “*keeping them busy*,” a “highly questionable” expedient particularly criticized in a South African study (Lopes et al., 2009). Here, additional schoolwork and nonschoolwork tasks like sweeping the floor were assigned to children with ADHD to prevent them from disrupting the lesson. As Lopes et al. (2009) note, similar practices can create disparities in the quality of educational experience learners receive and undermine their dignity and confidence to learn.

Extra schoolwork could also be aimed at strengthening learning. While this practice could benefit the learners in some respects, it could equally introduce discrimination between learners. Otukile-Mongwaketse et al. (2016) stressed that remedial classes were inappropriately used to implement strategies, such as slower pace and adaptations, which should rather be applied during normal classes, to the benefit of all learners. Otukile-Mongwaketse et al. (2016) commented that this violation of the whole-class principle was “not what inclusion is about.”

Similarly, grouping children per abilities with the aim to focus on learners who need the most support, or differentiate tasks, may also create unwanted distinctions across students, by preventing the focus on the whole class of children learning inclusively. If these separations are, or are perceived, as fixed, students considered weaker or struggling are likely to be discriminated against and to feel unable to improve, as pointed out by some of the authors (Mangope, 2017; Ngcobo & Muthukrishna, 2011; Yoro et al., 2020). A student in a lower-level group in a South African class attributed his lack of progress to the group they were part of:

*I do not want to belong to that group anymore. I did not even obtain a single correct answer*. (Ngcobo & Muthukrishna, 2011)

### Theme 4: Promotion of an Inclusive Environment

Teachers adopted a whole-class approach when promoting an inclusive environment, by organizing time and space and fostering peer inclusion. While teachers discussed the application of these strategies specifically for children with disabilities, they implemented them to the benefit of all learners, shaping the class environment as a whole.

#### Subtheme 4.1: Organized time and space

The studies supported the idea that children with DD benefit from an enabling classroom environment. Reducing distractions in the social and physical environment was considered important for the inclusion of children with ADHD (Seabi, 2010), autism (Majoko, 2017), and fetal-alcohol syndrome disorder (FASD) (Van Schalkwyk & Marais, 2017). To this end, teachers “*sensitised typically developing learners to maintain low levels of noise*” (Majoko, 2018) and tried “playing peaceful music” (Van Schalkwyk & Marais, 2017).

In one study on including learners with autism (Majoko, 2017), teachers seemed particularly concerned with environmental adaptations to minimize anxiety from excessive sensory stimulation. These included avoiding “*bright wall charts*” and creating “*safety zones*,” where learners could retreat away from intense sensory stimuli.

The school day was similarly organized through clear routines. Teachers reported that predictability reduced learners’ distractions and prevented anxiety, fostering inclusion of children with DD, especially autism and FASD, as well as benefiting those students without DD.

#### Subtheme 4.2: Peers’ inclusion

A second set of strategies used to foster inclusive environments promoted peer acceptance and positive interactions. For example, they fostered peers’ understanding and inclusion of learners with DD, by introducing “*disability issues*” (Majoko, 2017) in the curriculum.

Additionally, six studies reported that teachers purposely called children with DD to answer questions and help other students and gave them responsibilities and leadership roles in group activities, with the aim to empower them and foster peers’ acceptance.

*I make her group leader. I ask her to share and collect all their exercise books and pencils. I want the other children to know she can also do something . . . be their leader. So they obey her . . . it encourages her to come to school always*. (Okyere et al., 2019a)

More generally, teachers strived to foster a “collaborative culture” (Majoko, 2018) and a “friendly atmosphere” (Van Schalkwyk & Marais, 2017). They directly promoted peer interactions and organized whole-class cooperative activities to engage children with DD, encourage their “equal participation” (Yoro et al., 2020), and “improve their self-esteem” (Mangope, 2017).

With regards to group and pair work, Yoro et al. (2020) emphasized how “both methods develop learners’ oral communication and leadership skills.” Teachers in multiple studies considered social skills development an important step towards inclusion of children with DD and used cooperative learning as a way to enhance them. A few studies on including children with autism reported specific strategies to further ensure that learners interacted positively: encouraging peers to adapt their communication style, creating clear social rules, and reinforcing positive interactions.

### Theme 5: Engaging Teaching: Simple, Concrete, Interactive

Some of the most promising whole-class strategies used by teachers shared the aim to communicate and teach content in concrete and relatable ways, with simple communication, teaching aids, and selected teaching activities.

#### Subtheme 5.1: Simple communication

Six studies highlighted the need for clear language, “*straightforward instructions*” (Mangope, 2017), concise written summaries and thorough explanations, to foster learners’ understanding. Mokobane (2011) reported that in South Africa communication was simplified by teaching in the local home language, even when teaching in English was prescribed in policies.

It was stressed that inclusive communication and instruction should be targeted at individual needs of learners in the class and relatable, with examples and language drawn from learners’ daily life and interests. Two studies on the inclusion of children with autism (Majoko, 2017, 2018) reported that inclusive language avoids abstract concepts, uses concrete life examples and may be accompanied by visual communication through familiar gestures.

*I pair gestures that are used in his daily life with speech while teaching. I relate my teaching to his home and community life and daily experiences.* (Majoko, 2018)

#### Subtheme 5.2: Concrete teaching aids

As well as concrete examples, the hands-on approach employed by some teachers involved using concrete objects to convey abstract concepts. A student teacher reported telling a learner during a math lesson in South Africa:

*Okay fine, let’s just take out whatever you have in your pencil case and let’s try and work this out visually*. (Walton & Rusznyak, 2014)

A favorite strategy to enhance communication was the use of visual aids, mentioned in ten studies. For example, visual schedules with images describing school activities were used for learners with autism (Majoko, 2017, 2018, 2019) and FASD (Van Schalkwyk & Marais, 2017). Cards, posters, images, drawings, mind maps, slides, and other objects were used during lessons to promote inclusive teaching for all learners.

As well as supporting learners’ reasoning, understanding, and memory of taught material, another identified benefit of concrete and visual aids was enhancing their engagement, by keeping their focus and making the lesson more interesting.

One teacher made puppets out of cheap paper bags to encourage shy learners to get involved. (Potgieter-Groot et al., 2012)

#### Subtheme 5.3: Simple and interactive teaching activities

Teachers reported two complimentary teaching methods used for inclusive teaching: clear and slow direct instruction and an experiential, interactive approach.

Teachers in three studies reported the value of using direct instruction: A topic was introduced, explained clearly, and revised in the following session. A step-by-step approach and task analysis were frequently mentioned to organize learning effectively. More generally, teaching at a slower pace and repeating concepts was considered important to foster learning for children with difficulties. Paired with extra support, slower teaching pace was understood by teachers as the most straightforward way to support learners with higher learning needs.

The value of experiential teaching was also recognized. The numerous interactive activities teachers organized included simulations, debates, and collaborative problem-solving, to promote exploratory learning. Other fun and creative activities, such as playing and listening to music, art and craft, quizzes, games, storytelling, role-play, and drawing, were used to promote children’s engagement and enhance their learning of the school curriculum.

Group and pair work, previously highlighted for their benefits to peer inclusion, were considered another effective form of interactive teaching. Our analysis pointed at two main ways in which these activities promoted learning. First, they encouraged greater participation and engagement with the taught material. Secondly, peers could help explain the content to learners who were struggling and help them complete the tasks. Study authors and teachers particularly emphasized this second aspect with regards to peer tutoring, conducted by pairing one pupil “*who understands and one who doesn’t yet*” (Mangope, 2017). However, according to children with disabilities themselves, “any peer in class that was friendly to them could support them” (Bannink, Nalugya, & Van Hove, 2020).

### Theme 6: Fit With and Deviation From the Evidence Base

Several authors reflected on how the strategies teachers implemented fit in with previous research, demonstrating the assumption that teachers’ strategies should be evidence-based, often relatively to education theories developed in high-income countries. Teachers who received specific training appeared to engage in “more advanced strategies” (Potgieter-Groot et al., 2012) for teaching purposes, behavior management, and social skills training, as identified both by study authors’ reflections, and direct comparisons across studies during the current analysis. Less-trained teachers often learnt through experience to apply strategies broadly aligning with the evidence base. The use of strategies could be competent, creative, and organized, as outlined in the first subtheme, but also encounter challenges, described in the second subtheme. While Ugandan studies did not include reflections relevant to this theme, there were some cross-country differences in the reported fit with evidence base among other studies. In Ghana and Botswana, there was more emphasis on the difficulties faced in applying inclusive strategies. In Zimbabwe, Majoko (2017, 2018) highlighted teachers’ positive efforts and results in applying evidence-based strategies. Finally, several South African studies included relevant reflections, painting a more faceted picture, with both creative and inconsistent use of strategies.

#### Subtheme 6.1: Creative and organized use

Study authors and participants believed that successful inclusive education requires a creative and organized use of pedagogical strategies. For example, Potgieter-Groot et al. (2012) described teachers creating novel stories and games to meet learners’ needs, while Seabi (2010) reported that they combined strategies together for effective inclusion. An organized and creative use of strategies requires advance planning, as emphasized in three studies, which highlighted the importance of lesson plans for effective implementation of pedagogical strategies (Majoko, 2017; Mangope, 2017; Walton & Rusznyak, 2014). It was suggested that a lack of planning could result in missed opportunities to apply a strategy:

*I am not aware that I sometimes do not reinforce, maybe it is because I do not have that in my lesson plan*. (Mangope, 2017)

#### Subtheme 6.2: Inconsistent use

The quote in the previous theme exemplifies how teachers who employed inclusive strategies did not always do so consistently. Studies reported the limited use of several inclusive strategies. Mangope (2017), in Botswana, and Mokobane (2011), in South Africa, specifically discussed a range of strategies used inconsistently and emphasized this as a substantial barrier to inclusion.

Moreover, most teachers applied a small number of generic strategies that they knew to be inclusive (e.g., group work), rather than selecting specific strategies based on individual children’s needs. Mangope (2017) recommended that teachers should be enabled to use more advanced evidence-based techniques (e.g. task analysis and prompting), and select them accordingly to specific needs.

Appropriate implementation of pedagogical strategies was found to be further hindered by environmental challenges. The analysis clarified that some of the barriers to inclusive education discussed in Genovesi et al. (2022) were specifically limiting teachers’ ability to employ appropriate strategies. For example, due to the large class sizes of 40 to 80 children, providing extra attention to learners identified as weaker or struggling or individualized teaching was “an impossible task” (Mohamed & Laher, 2012).

*The classrooms are packed with children and it is very troubling to teach using any other method apart from the lecture method.* (Alhassan & Abosi, 2017)

## Discussion

While the African context is varied, inclusive education is in line with education policies in several African countries (Genovesi et al., 2022). However, education systems in these countries also present human and material resource shortages that may challenge inclusion. In this review, we explored how teachers strive to include children with DD despite these challenges. We analyzed extracts from 28 study reports concerning the pedagogical strategies and approaches teachers use in African schools to include children with DD. We developed six overarching themes, with subthemes closely interrelated across themes. Our analysis highlighted that teachers in multiple studies were responsive to individual needs, through curriculum and task adaptations and individualized instruction (Theme 1), as well as applying inclusive strategies to the whole class, rather than restricted groups of learners identified as weaker or struggling (Theme 3). When focusing specifically on the needs of children with DD, it was the agreement of participants throughout the studies that they required targeted learning support and extra attention through positive reinforcement (Theme 2). Themes more strongly related to Theme 3 (“Whole-Class Approach”) concerned building an inclusive environment (Theme 4) and the engaging and direct methods used for teaching the curriculum (Theme 5), as these were the areas in which teachers were most likely to focus on the class as a whole. Theme 6 highlighted how study authors often evaluated teachers’ use of strategies by comparing it to the evidence base, usually from high-income countries, and reported a creative and organized use at times, and others an inappropriate one, or too generic. Participants attributed limitations in strategy use to previously identified common challenges to inclusive education, such as large class sizes (Genovesi et al., 2022). Table 1 summarizes the strategies identified.

**Table 1:** Strategies identified

Strategies	Relevant Theme/Subtheme
Adapting taught content	Theme 1: Meeting Learners’ Needs
Teaching social and life skills	Theme 1: Meeting Learners’ Needs
Task differentiation with similar learning objectives	Theme 1: Meeting Learners’ Needs
Assessment adaptations (expanded opportunities and alternative response modes)	Theme 1: Meeting Learners’ Needs
Individualized education plans	Theme 1: Meeting Learners’ Needs
Extra individualized attention	Subtheme 2.1: Extra Support
Extra education support and remedial classes	Subtheme 2.1: Extra Support; Subtheme 3.2: Unhelpful Divisions
Immediate feedback	Subtheme 2.2: Immediate Feedback and Reinforcers
Reinforcers (praises, preferred activities and play, preferred objects and toys, and other tokens and rewards)	Subtheme 2.2: Immediate Feedback and Reinforcers
Ability grouping	Subtheme 1.1: Curriculum and Task Adaptations; Subtheme 2.1: Extra Support; Subtheme 3.2: Unhelpful Divisions
Environmental adaptations	Subtheme 4.1: Organized Time and Space
Sensitizing peers	Subtheme 4.1: Organized Time and Space; Subtheme 4.2: Peers’ Inclusion
Giving responsibilities	Subtheme 4.2: Peers’ Inclusion
Group work	Subtheme 1.1: Curriculum and Task Adaptations; Subtheme 4.2: Peers’ Inclusion; Subtheme 5.3: Simple and Interactive Teaching Activities
Pair work	Subtheme 4.2: Peers’ Inclusion; Subtheme 5.3: Simple and Interactive Teaching Activities
Straightforward instructions	Subtheme 5.1: Simple Communication
Use of local home language	Subtheme 5.1: Simple Communication
Relatable terms and examples	Subtheme 5.1: Simple Communication; Subtheme 1.2: Individualized Approaches
Use of accompanying gestures	Subtheme 5.1: Simple Communication;
Use of visual and concrete aids (visual schedules, cards, posters, images, drawings, mind maps, slides and other objects)	Subtheme 5.2: Concrete Teaching Aids; Subtheme 4.1: Organized Time and Space
Direct instruction	Subtheme 5.3: Simple and Interactive Teaching Activities
Slow pace and repetition	Subtheme 5.3: Simple and Interactive Teaching Activities
Experiential and interactive activities (simulations, debates and collaborative problem-solving, playing and listening to music, art and craft, quizzes, games, storytelling, role-play and drawing)	Subtheme 5.3: Simple and Interactive Teaching Activities

Some strategies discussed in the analysis are in line with Disabilities Studies in Education, while others move away from it, suggesting that this Western framework, though in line with overall views of inclusion previously evidenced in African countries (e.g., Majoko, 2018; J. A.McKenzie & Dalton, 2020), may offer important insights into African pedagogies, while at times being insufficient to interpret local understandings and address African needs. A key tenet of Disabilities Studies in Education is the focus on all learners, without distinctions driven by disability labels (Baglieri, Bejoian, et al., 2011; Baglieri & Shapiro, 2012; Baglieri, Valle, et al., 2011). An important framework in this context is Universal Design for Learning (UDL), an approach based on the assumption that all learners have unique learning needs and that therefore aims to provide multiple pathways to successful completion of learning outcomes by presenting the material in multiple ways and offering children multiple means of engaging with it and demonstrating their learning (Baglieri, Valle, et al., 2011; CAST, 2018; Hitchcock et al., 2002; J. A.McKenzie & Dalton, 2020). In this approach, barriers to learning are minimized as learners are given choices that align with their own learning needs, consequently advancing participation of all students, as advocated for in Disabilities Studies in Education (Baglieri, Valle, et al., 2011; CAST, 2018; Hitchcock et al., 2002; J. A.McKenzie & Dalton, 2020). Consistent with UDL recommendations, the studies reviewed reported diverse assessment opportunities and a variety of communication and teaching strategies and interactive activities as well as frontal sessions (i.e. with teachers’ presentation without children’s direct involvement), as described in Theme 5: “Engaging Teaching: Simple, Concrete, Interactive.” The focus on engagement is itself particularly relevant to UDL, as learning opportunities for all are given by providing multiple means for learners to engage with the curriculum (Baglieri, Valle, et al., 2011; CAST, 2018; Hitchcock et al., 2002). However, even within Theme 4, the use of some teaching methods, such as employing concrete teaching aids, was still reportedly limited to children with DD. At times, engaging teaching and relatable communication strategies were only used in remedial classes, as discussed in Subtheme 3.2 (“Unhelpful Divisions”). This analysis echoes evidence reviewed by J. A.McKenzie and Dalton (2020) on incomplete implementation of UDL, due to the current context in African countries, and the need for whole-school systemic changes and training programs for teachers, needs discussed also in Genovesi et al. (2022) as key for implementing inclusive education.

More broadly, the review also demonstrates a heavy presence in the qualitative African literature included of special education approaches, alongside UDL strategies: Curriculum adaptations and other individualized approaches are often only applied to children with disabilities, for example, through targeted individualized education plans, rather than offering a more flexible curriculum for all children. Special education practices are well outside the remits of Disabilities Studies in Education, and can be considered as violating principles of inclusion within this framework (Baglieri, Bejoian, et al., 2011; Baglieri, Valle, et al., 2011; Slee, 2001; Slee & Allan, 2001). Nonetheless, these practices can be valuable to provide support to children with DD, especially in environments where removal of barriers has not completely been achieved and children with disabilities need accommodations in order to successfully engage with the taught material ( J. A.McKenzie & Dalton, 2020). As such, in African contexts, UDL principles are complemented by special education practices that address specific contextual difficulties. For example, in terms of curriculum adaptation, in South Africa, the introduction of three curricula for children with DD of different severity was preferred to a previous attempt at implementing a flexible curriculum for all aligned with principles of UDL ( J.McKenzie, 2021). However, special education strategies require careful considerations to always be in the best interest of learners, and to meet individuals’ needs without overemphasizing differences. Reflecting on the three curricula in South Africa, one author of this review noticed how these could encourage the segregation of children using specific curricula to special classes and schools, and questions the processes of allocation to one curriculum and flexibility of movement between curricula ( J.McKenzie, 2021). Similarly, some practices of curriculum adaptations used in the studies reviewed, both in the narrow sense of “what is taught” and in the broader sense that includes the “how” (the strategies and aids used), were questionable and at times questioned by the authors of the included reports. For example, it was unclear whether the choice of a functional curriculum, prioritizing life and occupational skills, was usually targeted to actual learners’ needs or applied indiscriminately to all children with DD, under the assumption that they could not learn an academic curriculum. As highlighted in the “Unhelpful Divisions” subtheme (3.2), in one study, extra-academic activities were even assigned to keep learners busy rather than for their own benefit. The same subtheme also explored ways in which a heavily differentiated curriculum could promote segregation within the class, through ability grouping. This practice was widely criticized by authors of the reports reviewed who, similarly to J.McKenzie’s (2021) perspectives on the South African curricula, were concerned about the allocation based on disability labels and the lack of flexibility of movement across groups.

Importantly, when considering similar gaps and inconsistencies in teachers’ application of inclusive education strategies, we must acknowledge practical challenges reported in many settings in the African context, including little teacher training and resources and extremely large class sizes under the responsibility of one unaided teacher (Genovesi et al., 2022). The latter issue, in particular, was reported as a barrier even to the implementation of those strategies that teachers are able and willing to implement with little training and resources, such as providing individualized support (Alhassan & Abosi, 2017; Mohamed & Laher, 2012; Okyere et al., 2019a; Otukile-Mongwaketse et al., 2016). Ghanaian studies in this review, which did not identify any use of “unhelpful divisions,” are among the ones that most strongly reported challenges related to large class sizes, potentially highlighting the need for such divisions when these challenges are present. A literature study aimed to inform policy in Botswana reported that scholarly perspectives on the impact of class sizes on general student achievement are mixed (Bulawa et al., 2020). However, the highlighted value of smaller classes was to provide opportunities for teachers to improve their pedagogy and deviate from traditional lecture methods (Bulawa et al., 2020), an approach that both our theoretical framework (Baglieri, Valle, et al., 2011; Hitchcock et al., 2002; J. A.McKenzie & Dalton, 2020) and teacher participants in the studies we reviewed recognize as key to including children with disabilities. Consequently, aiming to address the issue of class sizes, as well as teacher training and teaching resources, is highly recommended to enable more fruitful implementation of inclusive pedagogical strategies.

Notably, there was variability across countries in the reported fit with the evidence base, likely reflecting both the contextual variety of African countries, and differences in study settings and authors’ judgment. Such differences must be acknowledged, to highlight the importance of adaptation and contextualization when adopting strategies from another African countries. First, in Ugandan studies, the focus was on indigenous stakeholders’ perspectives, rather than an existing evidence base. Difficulties to apply evidence-based strategies due to limited training and large class sizes were especially highlighted in Botswana and Ghana. Notably, as discussed elsewhere (Genovesi et al., 2022), the Ghanaian studies included in this review also reported corporal punishment, and its disproportionate use on children with disabilities (Okyere et al., 2019a, 2019b). While difficulties in Ghana may be related to its income level, none of these challenges were reported in the other lower middle-income country in this review, Zimbabwe. The reports from Zimbabwe were associated with a single author (Majoko, 2017, 2018) and focused specifically on the inclusion of children with autism, including in settings with small class sizes (Majoko, 2017): As such, they may not be directly comparable with the reports from Ghana. Moreover, barriers to applying inclusive strategies appear to be present at all income levels, as shown by studies from Botswana and South Africa in this review, as well as by evidence outside of Africa (e.g., Faragher et al., 2021; Lindsay et al., 2013; Roberts & Simpson, 2016). The information provided in the reviewed studies, often including a small sample for one or few schools, is not sufficient to establish what contextual and cultural factors may be key in creating cross-country variations in teachers’ ability to use inclusive strategies. Future cross-cultural research may explore this by evaluating practices in a larger number and variety of school settings.

A crucial point to be raised is the heavy prevalence of Western principles and practices previously reported in Western countries (e.g., Gunn & Delafield-Butt, 2016; Harrison et al, 2013) and paucity of references to indigenous approaches to inclusion in the studies reviewed. Recent perspectives in inclusive education call for the decolonization of practices and a deeper attention to approaches that arise from local understandings of disability, inclusion, and pedagogy (Karisa et al., 2021; Muthukrishna & Engelbrecht, 2018). These perspectives, also relevant to education practice more widely (e.g., Shahjahan et al., 2022), echo calls for research led by low- and middle-income countries (LMICs) and development efforts and partnerships in the global mental health field, relevant to interventions for psychosocial disabilities in LMICs (Breuer et al., 2020; Dalglish, 2020; Gautier et al., 2018; Kola et al., 2021). In the current review, several authors discussed strategies used by local teachers only in relation to Western education theories. Only two studies in Uganda explored inclusive education practices through the lens of indigenous frameworks of disability and inclusion (Bannink, Nalugya, et al., 2020; Bannink, Nalugya, & Van Hove 2020). These stressed the collective aspect of inclusion, suggesting that individual needs should be “addressed within a larger framework of ‘we’ as a class” (Bannink, Nalugya, et al., 2020). Importantly, practices arising directly from African contexts may be better equipped to navigate environmental challenges of these settings, such as the lack of resources. For example, a broader focus on whole-class approaches compared to individualized attention, in line with indigenous perspectives (Bannink, Nalugya, et al., 2020), may reduce the impact of challenges posed by large class sizes. Similarly, it has been suggested that a deeper engagement with families and understanding of their perspectives on their child’s education, in line with the strong role of families in education in some African communities, may support the development of more sustainable solutions (Karisa et al., 2021). Therefore, the need for teacher training may be best addressed through training programs based on local understandings and designed with stakeholders. For example, studies on Ugandan understanding of inclusive education in a framework of togetherness and kindness (Bannink, Nalugya, et al., 2020; Bannink, Nalugya, & Van Hove 2020) led to the collaborative development of a training program centered around peer-support among children and among teachers (Bannink, Nalugya, et al., 2020). However, more research on indigenous frameworks in other African countries should be conducted, to identify context-specific principles to be included in training programs.

Importantly, some of these principles may be relevant to high-income countries as well, especially in, but not limited to, low-resourced contexts. For example, addressing individual needs in the Ugandan framework of “we” (Bannink, Nalugya, at al., 2020) is highly relevant to moving from a focus on special education to UDL and the removal of all barriers, as advocated for in the Disabilities Studies in Education field. However, concepts of best practice may often be assumed to flow unidirectionally from high-income to LMICs (Breuer et al., 2020), where they are often adopted without cultural considerations, and it is unclear whether high-income countries may be open to learn from LMICs (Harris et al., 2017; Kola et al., 2021). Future qualitative studies on inclusive education for children with DD should aim to uncover indigenous perspectives and how these can reshape pedagogical practices that may currently be a legacy of colonization and ongoing power differentials that may often make knowledge from high-income countries expected to be indiscriminately applied to LMICs.

In particular, studies aiming at exploring indigenous frameworks of inclusive education and context-specific teacher training context and free current practices from any contextually inappropriate Western influences should employ participatory methods. Genuine engagement and participation was at the basis of the reviewed Ugandan studies mentioned above (Bannink, Nalugya, et al., 2020; Bannink, Nalugya, & Van Hove 2020), which recruited relevant stakeholders, including children with DD and their caregivers, collected data through interviews, interactive workshops, and by encouraging drawings and pictorial narrations, elicited subsequent feedback from participants on the analysis and finally developed new practices working in full collaboration with a team of stakeholders (Bannink, Nalugya, et al., 2020). More generally, researchers and organizations in relevant fields, including global mental health, education, and disability research, advocate for stakeholder inclusion in research and development, often suggesting the methods highlighted above (e.g., Eaton et al., 2011; Fletcher-Watson et al., 2019, 2021; Hoekstra et al., 2018; Pellicano, 2018; Poulsen et al., 2022; UNESCO Global Education Monitoring Report Team, 2020).

## Limitations

The transferability of the synthesis presented is limited by the selection of studies included in the original review (Genovesi et al., 2022), which revealed a high prevalence of research from South Africa, which is substantially better-resourced than most other sub-Saharan African countries (World Bank, 2023) and arguably the one most prominently influenced by Western cultures (van den Berghe, 1967). Similarly, the majority of the studies focused on practices in primary school setting and may have limited applicability to high school settings.

Importantly, the current synthesis focused on strategies for inclusion in school settings where children with DD were enrolled and teachers were striving to include them. Therefore, it gives a partial account of the education on offer for children with DD in Africa, only marginally touching on exclusion and challenges, such as stigma and lack of adequate human and material resources, that often mean that a large majority of children with DD in these settings are not accessing inclusive classes or even education of any kind (McKenzie et al., 2013). An account of such broader environmental factors is given in Genovesi et al. (2022). Nonetheless, the current synthesis highlighted how such factors affect pedagogical strategies, particularly in Subthemes 3.2 (“Unhelpful Divisions”) and 6.2 (“Inconsistent Use”).

## Conclusion

The synthesis presented highlighted that teachers in several schools in African countries where children with DD were enrolled applied UDL approaches in line with Disability Studies in Education as well as targeted special education strategies. Study authors and participants reflected on how teachers strived to apply various pedagogical practices for the inclusion of children with DD aimed at increasing their participation and learning, rather than just their physical presence in the classroom. However, teachers’ efforts to employ a whole-class approach and meet individual learners’ needs were not always consistent, as at times teachers shaped divisions in the class or selected special education strategies and curriculum content based on diagnoses and independently of the specific needs of individual children. Use of strategies could also be negatively affected by large class sizes and a lack of planning, training, and/or resources. Teachers’ inclusive practices may benefit from specialized pedagogy training. Ideally, we recommend that these should be based on research on indigenous inclusive education practices, which is currently lacking in peer-reviewed publications, but could be more aligned with local cultures and more suited than Western strategies to address context-prevalent challenges such as large class sizes. As such, future research should include qualitative studies aimed to uncover indigenous perspectives, as well as cross-country studies that can identify the influence of specific contextual differences on education practices in different countries.

## Supplemental Material

sj-docx-1-rer-10.3102_00346543241288247 – Supplemental material for Inclusive Strategies for Children With Developmental Disabilities in Mainstream Classrooms in African Countries: A Systematic Review of Stakeholder Experiences, Attitudes, and Perspectives Supplemental material, sj-docx-1-rer-10.3102_00346543241288247 for Inclusive Strategies for Children With Developmental Disabilities in Mainstream Classrooms in African Countries: A Systematic Review of Stakeholder Experiences, Attitudes, and Perspectives by Elisa Genovesi, Akhina Gaches, Judith McKenzie, Charlotte Hanlon and Rosa A. Hoekstra in Review of Educational Research

sj-docx-2-rer-10.3102_00346543241288247 – Supplemental material for Inclusive Strategies for Children With Developmental Disabilities in Mainstream Classrooms in African Countries: A Systematic Review of Stakeholder Experiences, Attitudes, and Perspectives Supplemental material, sj-docx-2-rer-10.3102_00346543241288247 for Inclusive Strategies for Children With Developmental Disabilities in Mainstream Classrooms in African Countries: A Systematic Review of Stakeholder Experiences, Attitudes, and Perspectives by Elisa Genovesi, Akhina Gaches, Judith McKenzie, Charlotte Hanlon and Rosa A. Hoekstra in Review of Educational Research

## Notes

For the purposes of open access, the author has applied a Creative Commons Attribution (CC BY) license to any Accepted Author Manuscript version arising from this submission. EG is supported by the UKRI Economic and Social Research Council through a London Interdisciplinary Social Science Doctoral Training Partnership (LISS DTP) studentship (ES/P000703/1). RAH and CH receive support from the National Institute of Health and Care Research (NIHR200842) using UK aid from the UK Government and CH through the NIHR Global Health Research Group on Homelessness and Mental Health in Africa (NIHR134325) using UK aid from the UK Government. The views expressed in this publication are those of the authors and not necessarily those of the NIHR or the Department of Health and Social Care. CH receives support from the Wellcome Trust through grants 222154/Z20/Z and 223615/Z/21/Z.
