# Supplementary material for: Inclusive Strategies for Children With Developmental Disabilities in Mainstream Classrooms in African Countries: A Systematic Review of Stakeholder Experiences, Attitudes, and Perspectives
Source: Rev Educ Res. 2024 Oct 31;95(6):1183–212. doi: 10.3102/00346543241288247 (PMC12598068; doi:10.3102/00346543241288247)
Supplement: sj-docx-2-rer-10.3102_00346543241288247 – Supplemental material for Inclusive Strategies for Children With Developmental Disabilities in Mainstream Classrooms in African Countries: A Systematic Review of Stakeholder Experiences, Attitudes, and Perspectives [file sj-docx-2-rer-10.3102_00346543241288247.docx]

**Supplementary Material: point-by-point explanation of connections shown in the thematic map**

**Coloured arrows (A,B,C,D,E,F): connections across themes**

Among the connections across themes, arrows A and B (blue and red horizontal) highlight how meeting learners’ individual needs and employing a whole-class approach are two highly interrelated principles of IE: IE is about addressing each learners’ needs in the class, and therefore requires addressing individual needs, while also focusing on the whole class without making distinctions between children with and without disabilities. However, strategies aimed at promoting an inclusive environment and at teaching with simple, concrete, interactive methods are more reflective of a whole-class approach (C & D), while targeted support, reinforcement and feedback are more directed towards meeting individual needs (E). F shows how the final theme (Fit with and deviation from the evidence base) is relevant to the use of strategies discussed in all other themes.

**Black lines (g,h,I,j,k,l,m): connections across subthemes**

g: simpler and more concrete communication (more generally highlighted as a whole-class strategy) should be targeted at individual needs of learners. The communication style should be accessible and relatable, with examples and language drawn from learners’ daily life and interests.

h: diverse and simpler modes of communication are also relevant for task adaptations, as some of these adaptations directly involved allowing for diverse communication and giving learners opportunities for oral, rather than written, assessment, or to respond through drawings.

i and j: common barriers to the use of a whole-class approach and causes of unhelpful division were teachers’ attempts at providing adaptations or extra support to children with DD.

k: teachers reported accounting for children’s individual needs for organised environments when deciding how much structure to provide to learners.

l: extra support and slower teaching pace were often paired in reports and understood by teachers as the most straightforward way to support learners with higher learning needs.

m: some of the interactive teaching activities, especially cooperative ones, were also used to promote a collaborative environment in the class.
